# Altering Early Life Gut Microbiota Has Long-Term Effect on Immune System and Hypertension in Spontaneously Hypertensive Rats

**DOI:** 10.3389/fphys.2021.752924

**Published:** 2021-10-29

**Authors:** Francois M. Abboud, Michael Z. Cicha, Aaron Ericsson, Mark W. Chapleau, Madhu V. Singh

**Affiliations:** ^1^Abboud Cardiovascular Research Center, Carver College of Medicine, University of Iowa, Iowa City, IA, United States; ^2^Department of Internal Medicine, Carver College of Medicine, University of Iowa, Iowa City, IA, United States; ^3^Department of Molecular Physiology and Biophysics, Carver College of Medicine, University of Iowa, Iowa City, IA, United States; ^4^Department of Internal Medicine, Division of Endocrinology and Metabolism, Carver College of Medicine, University of Iowa, Iowa City, IA, United States; ^5^Metagenomics Center, University of Missouri, Columbia, MO, United States

**Keywords:** microbiome, immune system, gut microbiome, hypertension, SHR (spontaneously hypertensive rat), T helper (T) 17 cells, RORgamma t

## Abstract

Hypertension is regulated by immunological components. Spontaneously hypertensive rats (SHR) display a large population of proinflammatory CD161 + immune cells. We investigated the effect of early post-natal gut microbiota on the development of the immune system and resulting hypertension in the SHR. We first examined the microbial populations in the fecal samples of SHR and normotensive control WKY using 16S rDNA sequencing. We found that in the newborn SHR (1-week old) the gut microbiota was qualitatively and quantitatively different from the newborns of normotensive WKY. The representation of the predominant bacterial phylum Proteobacteria was significantly less in 1-week old SHR pups than in WKY (94.5% Proteobacteria in WKY vs. 65.2% in SHR neonates). Even within the phylum Proteobacteria, the colonizing genera in WKY and SHR differed dramatically. Whereas WKY microbiota was predominantly comprised of *Escherichia-Shigella*, SHR microbiota was represented by other taxa of *Enterobacteriaceae* and *Pasteurellaceae*. In contrast, the representation of phylum Firmicutes in the neonatal SHR gut was greater than WKY. Cross-fostering newborn SHR pups by lactating WKY dams caused a dramatic shift in 1-week old cross-fostered SHR gut microbiota. The two major bacterial taxa of phylum Proteobacteria, *Enterobacteriaceae and Pasteurellaceae* as well as *Lactobacillus intestinalis, Proteus, Romboustia* and *Rothia* were depleted after cross-fostering and were replaced by the predominant genera of WKY (*Escherichia-Shigella*). A proinflammatory IL-17F producing CD161 + immune cell population in the spleen and aorta of cross-fostered SHR was also reduced (30.7% in self-fostered SHR vs. 12.6% in cross-fostered SHR at 30 weeks of age) as was the systolic blood pressure in adult cross-fostered SHR at 10 weeks of age. Thus, altered composition of gut microbiota of SHR toward WKY at early neonatal age had a long-lasting effect on immune system by reducing proinflammatory immune cells and lowering systolic blood pressure.

## Introduction

Gut microbes exert profound effects on health and disease by assisting in development of the gut, maintaining gut barrier function, providing energy and protecting against pathogenic invasion. The early neonatal gut microbiome primes the development of the host immune system (Hooper et al., 2012; Gensollen et al., 2016; Li et al., 2017) with potentially long-lasting health consequences in a manner dependent on the genetic background of the host (Macpherson et al., 2017). The composition of the early gut microbiome is dependent on host genetic factors and the environment (Turnbaugh et al., 2009; Benson et al., 2010) and can be altered by changing the environment at an early post-natal age (Daft et al., 2015).

Hypertension is associated with neurogenic, environmental, and immunologic factors. The spontaneously hypertensive rat (SHR) is a widely studied model of genetic hypertension that is linked to both immune disorder (Singh et al., 2017) and “unhealthy” gut microbiome (dysbiosis) (Yang et al., 2015). Treatment of SHR with antibiotics or fecal transplant from normotensive rats reduces blood pressure suggesting a role of resident gut microbiota in SHR hypertension (Galla et al., 2018). These results indicate a potential interaction of the immune system and gut microbiome in SHR hypertension. Effects of gut microbiome on adult blood pressure in other models have been reported. Use of angiotensin II (Ang II) was correlated with change in gut microbiome (Yang et al., 2015), oral administration of minocycline antibiotic reduced blood pressure presumably by altering the gut microbiome and fecal transplants from normotensive rats to stroke-prone SHR (SHRSP) reduced blood pressure (Adnan et al., 2017). However, these models have inherent limitations; considering the quick effect of Ang II on hypertension, it is not clear whether in Ang II-induced hypertension the change in gut microbiota was a consequence of Ang II administration. Minocycline antibiotic crosses the blood-brain barrier (Shi et al., 2010; Hu et al., 2013) and can have direct neurogenic effects in hypertension, whereas SHRSP is a distinct strain of rat that genetically diverged considerably from SHR (Nabika et al., 2012). Moreover, late age colonization of the gut by introduced microbiota does not fully restore the immune system in germ free mice (El Aidy et al., 2013). Therefore, it appears that gut microbiome may affect hypertension at different stages through different mechanisms.

We and others have used animal models to show the roles of pro-inflammatory immune cells in development of hypertension. We have shown that the SHR displays an abnormally high preponderance of CD161 + immune cells at birth that increases with age (Singh et al., 2017). These cells also express high levels of RORγt transcription factor-dependent proinflammatory prohypertensive cytokines IL-17A and IL-17F that contribute to hypertension (Singh et al., 2017). IL-17A and -17F are members of the IL-17 family of proinflammatory cytokines that are produced by T helper 17 (Th17) cells, γδT cells, and other cells that express RORγt transcription factor (Ivanov et al., 2006). Th17 cells play a prominent role in several autoimmune diseases and their abundance is regulated by specific gut microbiota (Ivanov et al., 2008; Eberl, 2017).

In the SHR, significantly lower blood pressure levels were observed in adulthood, when the neonatal pups were cross-fostered by normotensive Wistar-Kyoto (WKY) or Sprague-Dawley dams (Cierpial and McCarty, 1987). Although, the outcome of cross-fostering on SHR hypertension has been attributed to altered maternal behavior, we hypothesized that cross-fostering alters the gut microbiota of the neonatal SHR resulting in a favorable immune-response and lesser hypertension in adulthood. Indeed, it is known that the development of the immune system can be affected by gut microbiota as early as in the fetal stage (Cierpial et al., 1988; Mishra et al., 2021). Here, we investigated whether altering the neonatal gut microbiota of the SHR pups by cross fostering with normotensive dams favorably alters their immune response and their hypertension in adulthood.

## Materials and Methods

### Animal Breeding and Cross-Fostering

All animal protocols were approved by the Institutional Animal Care and Use Committee of the University of Iowa. WKY and SHR rats were obtained from Charles River Laboratories and were bred in-house. All rats were maintained on a standard rat chow diet (Cat # 7013, Envigo, United States). Cages of pregnant dams (1 dam per cage) were monitored daily, and the day of the pups’ birth was considered day-1. Pups were weaned at 3 weeks of age. Pups of WKY and SHR strains were cross-fostered within 48 h of birth by presenting the entire litter (8 to 10 pups each litter) to the cages of the fostering dams of the other strain. Only male pups were used in the experiments.

### Fecal Samples and DNA Extraction

Fecal or colonic samples were obtained from 1 week old, 5 weeks old and 12 weeks old (equivalent to early adulthood in humans) male rats. All fecal samples were obtained in the mornings. For 1 week old pups, colonic samples were obtained by decapitation of pups under anesthesia (isoflurane) and exposure of the body cavity and gentle extrusion of fecal matter using sterile blunt forceps. For older rats, fecal samples, free from urine, were obtained by placing the rats in sterilized cages and pellets were collected on first appearance. All samples were handled using sterile forceps and stored in sterile screw-cap tubes by quick freezing in liquid nitrogen, followed by transfer to −80°C until further analyses. DNA was extracted using PowerFecal kits (Qiagen) according to the manufacturer’s instructions, with the exception that samples were homogenized in the provided bead tubes using a TissueLyser II (Qiagen, Venlo, Netherlands) for 3 min at 30 beats/sec. DNA yields were quantified by fluorometry (Qubit 2.0, Invitrogen, Carlsbad, CA, United States) using quant-iT BR dsDNA reagent kits (Invitrogen) and normalized to a uniform concentration and volume.

### 16S rRNA Gene Sequencing

16S rRNA amplicon libraries were generated and sequenced at the MU Genomics Technologies Core facility. Bacterial 16S rRNA amplicons were constructed via amplification of the V4 region of the 16S rRNA gene with universal primers (U515F/806R) previously developed against the V4 region, flanked by Illumina standard adapter sequences (Caporaso et al., 2011; Walters et al., 2011). Oligonucleotide sequences are available at proBase (Loy et al., 2007). Dual-indexed forward and reverse primers were used in all reactions. PCR was performed in 50 μL reactions containing 100 ng metagenomic DNA, primers (0.2 μM each), dNTPs (200 μM each), and Phusion high-fidelity DNA polymerase (1U, Thermo Fisher). Amplification parameters were 98°C(^3 *min*^) + [98°C(^15 *sec*^) + 50°C(^30 *sec*^) + 72°C(^30 *sec*^)] × 25 cycles + 72°C(^7 *min*^). Amplicon pools (5 μL/reaction) were combined, thoroughly mixed, and then purified by addition of Axygen Axyprep Mag PCR clean-up beads (Corning, United States) to an equal volume of 50 μL of amplicons and incubated for 15 min at room temperature. Products were then washed multiple times with 80% ethanol and the dried pellet was resuspended in 32.5 μL EB buffer (Qiagen), incubated for 2 min at room temperature, and then placed on the magnetic stand for 5 min. The final amplicon pool was evaluated using the Advanced Analytical Fragment Analyzer automated electrophoresis system, quantified using quant-iT HS dsDNA reagent kits, and diluted according to Illumina’s standard protocol for sequencing on the MiSeq instrument as 2 × 250 bp paired-end reads.

### Microbiome Analysis

DNA sequences were assembled and annotated at the MU Informatics Research Core facility. Primers were designed to match the 5′ ends of the forward and reverse reads. Cutadapt (Martin, 2011) (version 2.6^1^) was used to remove the primer from the 5′ end of the forward read. If found, the reverse complement of the primer to the reverse read was then removed from the forward read as were all bases downstream. Thus, a forward read could be trimmed at both ends if the insert was shorter than the amplicon length. The same approach was used on the reverse read, but with the primers in the opposite roles. Read pairs were rejected if one read or the other did not match a 5′ primer, and an error-rate of 0.1 was allowed. Two passes were made over each read to ensure removal of the second primer. A minimal overlap of three bp with the 3′ end of the primer sequence was required for removal.

The QIIME2 (Bolyen et al., 2019) DADA2 (Callahan et al., 2016) plugin (version 1.10.0) was used to denoise, de-replicate, and count ASVs (amplicon sequence variants), incorporating the following parameters: (1) forward and reverse reads were truncated to 150 bases, (2) forward and reverse reads with number of expected errors higher than 2.0 were discarded, and (3) Chimeras were detected using the “consensus” method and removed. R version 3.5.1 and Biom version 2.1.7 were used in QIIME2. Taxonomies were assigned to final sequences using the Silva.v132 (Pruesse et al., 2007) database, using the classify-sklearn procedure. Rarefaction of data was performed using https://docs.qiime2.org/2021.8/plugins/available/diversity/core-metrics-phylogenetic/. In summary, data were rarefied to one less than the lowest read count greater than 10,000 reads. Alpha (Shannon diversity index) and beta-diversity (Bray-Curtis similarities) were calculated and principal coordinate analysis plots were generated using Past3 software.

### Tissue Processing for Flow Cytometry

Rats were euthanized under anesthesia and single cell suspensions from spleens were obtained by disaggregating spleen tissue between frosted glass microscopy slides. Erythrocytes were lysed by suspending splenocytes pellets in a hypotonic solution (155 mM NH4Cl, 12 mM NaHCO3, 0.1 mM EDTA), nucleated cells were washed twice with phosphate buffered saline (PBS). Flow cytometry was performed as described earlier (Singh et al., 2017). Briefly, washed splenocytes were suspended in Fc blocking buffer (2% v/v fetal bovine serum and 1% v/v normal mouse serum in PBS). An aliquot of 10^6^ cells was taken from each sample and mixed with FITC-CD161, PerCP-CD8a, APC-CD4, PE-CD3, and PE-Cy5-CD45RA antibodies (1 μl each antibody, all antibodies from BD Biosciences). After incubation with antibodies on ice for 30 min, cells were washed twice with PBS and resuspended in 400 μl PBS and flow cytometry was performed on a Beckton-Dickinson Aria flow cytometer. Thoracic aorta were minced with a razor blade and digested in 0.05% w/v Collagenase Type I and 0.05% w/v Collagenase Type II in HBSS. Cells were dissociated by trituration and filtered through a nylon membrane followed by two washes with ice cold PBS. Cells were then processed as described above for the spleen cells.

### Tail Cuff Pressure Measurements

Tail cuff pressure measurements were done as previously described (Singh et al., 2019). Briefly, a week prior to recordings, rats were acclimatized in the holders without tail cuff pressure measurements for at least three sessions. Systolic blood pressure (SBP) was recorded every week by tail cuff plethysmography on a VisiTech 2000 system (VisiTech, Denver, CO, United States). Weekly SBPs reported were averages of 20 recordings per animal per session each week. Statistical analyses of SBPs were performed with a two-way ANOVA (Prism Software, version 7.0a; GraphPad Software, La Jolla, CA, United States) on SBP data starting from 6 weeks of age, when the increase in pressure in SHR became evident. Investigators were blinded for blood pressure and microbiome analyses.

### Statistical Tests

Statistical tests were performed using GraphPad Prism. Results are presented as mean ± SEM. For comparisons between WKY and SHR or between self-fostered and cross-fostered groups, one-way ANOVA with Sidak’s *post hoc* test was applied for comparisons between multiple groups. For blood pressure, a two-way ANOVA was used. *P* values < 0.05 were considered statistically significant.

## Results

We examined the gut microbiome using 16S rDNA sequencing from the fecal samples of neonatal (1 week), of weanlings (5 weeks), and of adult (12 weeks) male SHR and WKY rats. Two major comparisons were made. One was between neonatal rats and older (weanlings and adults) rats. The other was between the two strains, SHR and WKY.

**FIGURE 1:**
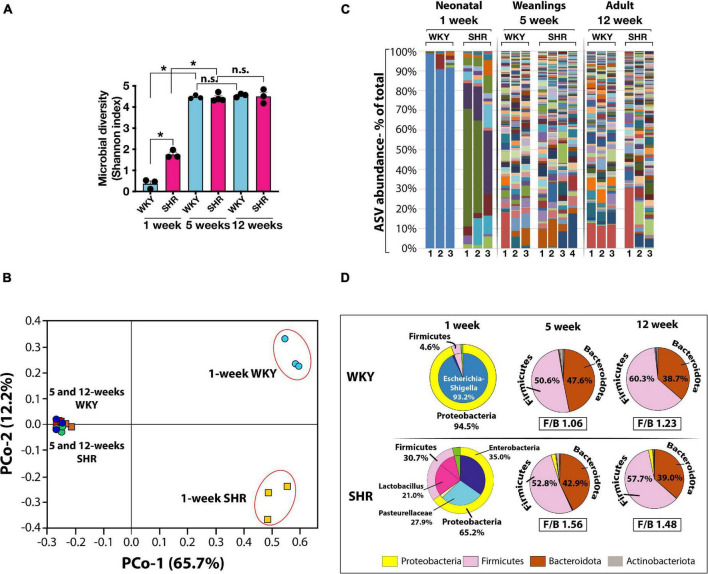
**(A)** Microbial diversity (Shannon Index) and complexity are significantly greater in weanling (5-weeks old) and adult (12-weeks old) WKY (blue bars) and SHR (red bars) compared to neonatal (1-week old) rats. (*n* = 3 each group except *n* = 4 in 5-weeks old SHR). Asterisks indicate significant difference (*P < 0.0001). **(B)** Principal component (PCo) analysis of clustering of microbiotas is also very different in neonatal 1-week old than older rats (PCo1, *x*-axis) and between 1-week old WKY and SHR (PCo2, *y*-axis). **(C)** Graphical display of major ASV (Amplicon Sequence Variants) in WKY and SHR from 1-week (*n* = 3 each), 5-weeks (WKY *n* = 3, SHR *n* = 4) and 12-weeks old (*n* = 3 each) rats. Different taxa are displayed in different colors and the height of each colored bar represents the population size (percent of total population). Each lane represents one sample from a mouse. **(D)** Pie charts display the major phyla and constituent ASV at different ages in WKY and SHR (*n* = 3 in each group except *n* = 4 in 5-weeks old SHR). Corresponding Firmicutes to Bacteroidota ratio (F/B ratio) are shown for 5- and 12- weeks old rats. F/B ratios for 1-week old rats were not calculated due to extremely low content of Bacteroidota taxa.

In the neonates of both strains, the number of microbial species (Amplicon Sequence Variants, ASV) and their complexity (Shannon index) were significantly less than in the weanlings and adult counterparts (Figures 1A,B). No major difference between the microbiome of the older rats of the two strains were seen (Figures 1B,C). In the neonates, the phylum Proteobacteria was more dominant in WKY than in SHR (Figure 1D) and consisted primarily of *Escherichia-Shigella* (93.2%), whereas in neonatal SHR there was a total lack of *Escherichia-Shigella*, and instead the Proteobacteria of *Enterobacteriaceae* and *Pasteurellaceae* prevailed at 35.0 and 27.9%, respectively (Figure 1D). In contrast, the phylum Firmicutes was more prevalent in SHR (30.7%) than in WKY (4.6%) with *Lactobacillus* constituting 21.0% in SHR and essentially lacking in WKY (0.86%, Figure 1D).

In the weanlings and adults of both strains the phylum Proteobacteria was notably absent, but the Firmicutes was significantly and equally represented as was the phylum Bacteroidota (Figure 1D). Thus, the major difference between the strains was in their neonatal state. The Proteobacteria *Escherichia-Shigella* was absent in neonatal SHR, yet dominant in WKY and the Firmicutes *Lactobacillus* present in SHR was absent in WKY. The Firmicutes to Bacteroidota ratio (F/B ratio) in 5-weeks old WKY and SHR were 1.06 and 1.23, respectively (Figure 1D). The F/B ratio in 12-weeks old WKY and SHR were also similar (WKY 1.56 and SHR 1.48).

**FIGURE 2:**
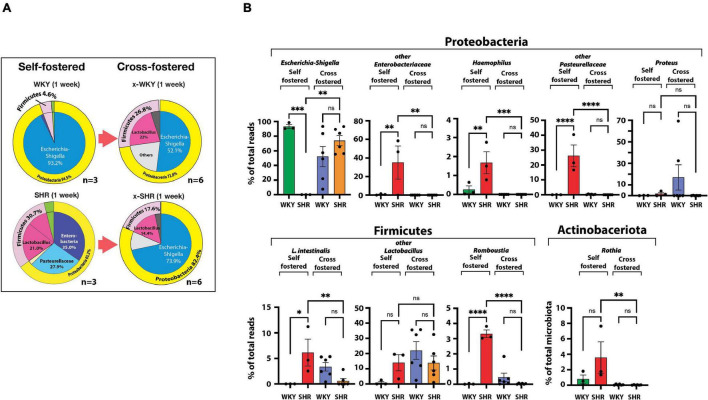
**(A)** Differences in neonatal major phyla between self-fostered (*n* = 3 each) and cross-fostered x-WKY and x-SHR (*n* = 6 each). **(B)** Changes in major ASV of the gut microbiota between 1-week old self-fostered WKY and SHR (*n* = 3 each) and between 1-week old cross-fostered x-WKY and x-SHR (*n* = 6 each). Asterisks denote statistically significant difference (^∗^*P* < 0.05, ^∗∗^*P* < 0.01, ^∗∗∗^*P* < 0.001, ****P ≤ 0.0001, one-way ANOVA with Sidak’s *post hoc* multiple comparison test), n.s. denotes not statistically significant.

We then cross-fostered newborn SHR and WKY pups with WKY and SHR dams, respectively. Cross-fostering of SHR dramatically shifted their Proteobacteria to resemble the distribution seen in self-fostered WKY. *Escherichia-Shigella*, which is essentially absent in the self-fostered neonatal SHR, increased to 73.9% by cross-fostering (Figure 2A) replacing *Enterobacteriaceae* and *Pasteurellaceae* taxa that are prominent in neonatal SHR and dropped to undetectable levels with cross-fostering (Figure 2A).

Equally significant changes were observed in cross-fostered WKY with a reduction in the Proteobacteria *Escherichia-Shigella* from the level of 93.2% seen in self-fostered WKY to 52.1% in the cross-fostered, and the new appearance of the Firmicutes *Lactobacillus* in the cross-fostered WKY to 22.0%, a level comparable to the self-fostered SHR (21.0%). The significant differences of the major taxa, which constitute approximately 98% of total taxa between 1-week old self-fostered WKY and self-fostered SHR, were abolished in cross-fostered WKY and cross-fostered SHR (Figure 2B). Comparison of these groups showed elimination of *Haemophilus*, *Lactobacillus intestinalis* (*L. intestinalis*), *Romboustia*, and *Rothia* in cross-fostered SHR (Figure 2B). In addition, *Enterobacteriaceae* and *Pasteurellaceae* taxa were also eliminated in cross-fostered SHR. In contrast, cross-fostered SHR showed increased *Escherichia-Shigella*.

**FIGURE 3:**
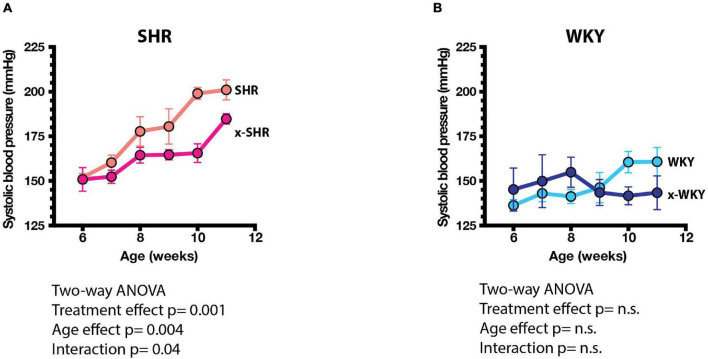
**(A)** Systolic blood pressures (SBP) of self-fostered SHR vs. cross-fostered SHR, and **(B)** self-fostered vs. cross-fostered WKY at different ages. SBP was significantly lower in cross-fostered than self-fostered SHR. Results were analyzed by two-way ANOVA as shown.

The systolic blood pressure measured by tail cuff method increased progressively more in self-fostered SHR than in cross-fostered SHR, and was significantly lower in cross-fostered adult SHR (Figure 3A) similar to the previous report (Cierpial and McCarty, 1987). The systolic blood pressure of self-fostered and cross fostered WKY did not increase with age and did not differ between the groups (Figure 3B).

**FIGURE 4:**
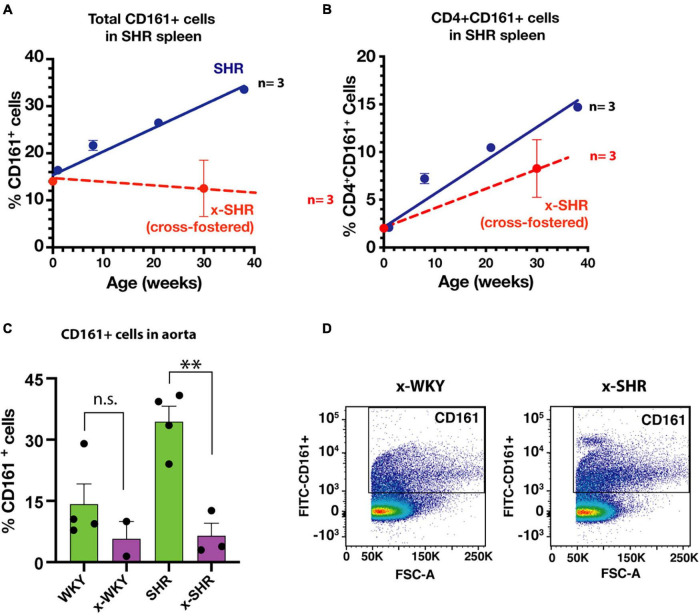
**(A)** Cross-fostering of SHR prevents the age dependent increase in CD161 + cells and **(B)** CD4 + CD161 + cells observed in self-fostered SHR as previously reported (Singh et al., 2017) (Blue line). **(C)** Comparison of infiltration of proinflammatory CD161 + immune cells in aortic tissue in self-fostered cross-fostered WKY and SHR. **(D)** Representative dot plot of CD161 + cells in thoracic aorta of cross-fostered WKY (x-WKY) and cross-fostered SHR (x-SHR). Asterisks denote statistically significant (^∗∗^*P* < 0.0075), whereas n.s. denotes statistically not significant results by one-way ANOVA and Sidak’s *post hoc* test.

We have previously reported in SHR a post-natal progressive increase in proinflammatory immune cells that express a CD161 surface marker with age. These cells produce IL-17F that influences the blood pressure in SHR (Singh et al., 2017). In this study, the splenic population of CD161 + cells in cross-fostered SHR did not increase with age when compared to the self-fostered SHR (Figure 4A). Similarly, CD4 + CD161 + cells, known to overexpress RORγt transcription factor and increase IL-17F cytokine upon induction, were also decreased (Figure 4B). Moreover, of pathological significance, infiltration of CD161 + cells in aortic tissue of cross-fostered SHR was also decreased (Figure 4C) and was similar to that observed in cross-fostered WKY (Figures 4C,D).

## Discussion

In this report, using SHR as a genetic model of hypertension, we have shown that altering gut microbiome of the neonatal SHR results in long term immune alteration and attenuated hypertension. Our 16S rDNA sequence data suggest that neonatal SHR gut microbiome is dramatically different from that of neonatal WKY much before the onset of hypertension. However, remarkable changes occurred as these rats from either strain aged. First, the number of bacterial taxa increase after weaning and second, the differences between the microbiome of the two strains diminishes. Indeed, similar results of low complexity of gut microbiome in infancy and increased microbial diversity with shift to solid food from mother’s milk have been reported in humans in longitudinal as well as population studies (Koenig et al., 2011; Yatsunenko et al., 2012; Arrieta et al., 2014). In addition, in the neonatal gut the taxa representing the order *Enterobacteriales* are predominant (Arrieta et al., 2014; Tsukuda et al., 2021), an observation that is consistent in our data of neonatal WKY. In the neonatal WKY the *Enterobacteriaceae* family was predominantly represented by *Escherichia-Shigella*, whereas this taxon was almost completely absent in neonatal SHR. Instead, other taxa of *Enterobacteriaceae* family were detected in the neonatal SHR. This is a remarkable deviation in SHR gut microbiome from normotensive WKY.

Age-related increase in the taxa of gut bacteria is usually accompanied with the increase in Firmicutes. We observed increased Firmicutes in both WKY and SHR with age. However, the Firmicutes population in neonatal gut was much greater in SHR than in neonatal WKY. A higher Firmicutes to Bacteroidetes ratio (F/B ratio) is considered to be a hallmark of hypertension (Yang et al., 2015; Zhang et al., 2021). Indeed, in our study, the neonatal SHR had much greater F/B ratio than neonatal WKY, primarily because early neonatal gut is devoid of Bacteroidota taxa. Nevertheless, this difference was increased in cross-fostered SHR suggesting that F/B ratio alone is not a reliable predictor of a disease state (Magne et al., 2020).

We observed major differences in several bacterial genera between the two strains. First, the neonatal WKY had predominant presence *of Escherichia-Shigella* whereas these bacteria were absent in SHR. In contrast, neonatal SHR had predominantly *Haemophilus*, *Lactobacillus intestinalis, Romboustia, Rothia and other taxa of Enterobacteriaceae, Pasteurellaceae*, and *Lactobacilli.* More importantly, cross-fostering of SHR by normotensive WKY dams significantly increased *Escherichia-Shigella* in 1-week old SHR, reduced *Lactobacillus* and almost completely depleted taxa of *Enterobacteriaceae*, *Pasteurellaceae*, *Romboustia*, and *Rothia*. These changes were associated with attenuated hypertension and decreased CD4 + CD161 + cells that produce high levels of IL-17F cytokine on induction (Singh et al., 2017) in adulthood.

We found a decrease in proinflammatory CD161 + cells in spleen as well as in aorta of the cross-fostered SHR. The decrease in systolic blood pressure of cross-fostered SHR at adulthood associates with the decrease in this cell population suggesting that the gut microbiome influences genetic hypertension through immunological development. Development and maturation of the mammalian immune system predominantly depends on the early neonatal interactions with the gut colonizing microbiota (Gensollen et al., 2016). We have previously demonstrated that the population of proinflammatory immune cells (CD161 + RORγt +) in SHR express IL-17F to influence the vasculature to influence blood pressure in SHR (Singh et al., 2017). Proliferation and abundance of IL-17 producing Th17 cells has been shown to be dependent on the presence of segmented filamentous bacteria (SFB, *Candidatus arthromitus*) in the gut (Ivanov et al., 2009; Ericsson et al., 2014). In our study, there was undetectable SFB in the neonatal (1 week old) pups of either SHR or WKY strain, which did not change after cross-fostering. However, SFB abundance was high in both WKY and SHR after weaning (5-weeks old), which declined in WKY more than in SHR at 12-weeks of age. It is likely that one or more of the bacteria present in neonatal SHR that were eliminated in cross-fostered SHR could contribute to the increase in the CD161 + immune cells leading to hypertension.

Mechanistically, gut microbe-host interaction is of key importance in establishing healthy gut barrier function, host-tolerance to commensal bacteria, and retrieval of metabolic products from the gut microbes. In addition to direct interaction between microbes and host mucosal cells, several nutritionally important metabolites produced by these commensal bacteria influence the health of the host (El Aidy et al., 2013; Mishra et al., 2021; Tsukuda et al., 2021). Gut bacteria produce short chain fatty acids (Correa-Oliveira et al., 2016; Natarajan et al., 2016; Li et al., 2018; Bartolomaeus et al., 2019) (SCFA) and trimethylamine-3-oxide (Jaworska et al., 2017) (TMAO) that are utilized as nutrients as well as serving as ligands for specific receptors (Macia et al., 2015) on host cells to profoundly affect the immune response and hypertension. We also observed that a number of previously reported SCFA-producing bacteria of SHR (Yang et al., 2015) were not detected in our study. However, SHR gut microbiome contains a high number of SCFA producing *Lactobacillus* (Koopman et al., 2019). Our study does not address the underlying mechanism of metabolites or direct microbe-host cell interaction, but it does identify *Haemophilus, L. intestinalis, Proteus, Romboustia*, and *Rothia* as potential bacteria that might be contributing to SHR hypertension.

The blood pressure in WKY that were cross-fostered by hypertensive SHR dams was not increased. Our results are consistent with previous cross-fostering studies (Cierpial et al., 1988). These findings are also consistent with a genetic propensity for hypertension in the SHR compared to WKY (Yamamoto et al., 2013). Despite the changes in the microbiome of WKY that were cross-fostered by SHR, these changes, in the absence of the genetic predisposition, were not sufficient to cause hypertension. In summary, we conclude that a remarkable difference between the gut microbiome of neonatal SHR and WKY exists; this difference diminishes into adulthood and yet has a lasting effect on the immune response and the development of hypertension. More importantly, cross-fostering of SHR with WKY changes the neonatal gut microbiome of SHR to reflect that of the WKY and has measurable long term favorable outcomes in reducing the inflammation and the level of hypertension in adult SHR. Although fecal microbiota transplant (FMT) in adult SHR reduces the inflammatory response and hypertension (Toral et al., 2019), our results show an additional different role of the early neonatal gut microbiota in development of hypertension in adulthood by modulating the long-term immune response.

## Data Availability Statement

The datasets presented in this study can be found in online repositories. The names of the repository/repositories and accession number(s) can be found below: NCBI (accession: PRJNA763085).

## Ethics Statement

The animal study was reviewed and approved by IACUC, University of Iowa.

## Author Contributions

FA contributed to conception of the study and writing the manuscript. MCi recorded blood pressure and assisted in all other experiments. AE performed the sequencing and statistical analysis. MCh contributed to the design of the study and revised and edited the manuscript. MS contributed to the conception and design of the study, performed experiments, wrote the first draft of the manuscript, analyzed data, and prepared the final draft. All authors contributed to manuscript revision, read, and approved the submitted version.

## Conflict of Interest

The authors declare that the research was conducted in the absence of any commercial or financial relationships that could be construed as a potential conflict of interest.

## Publisher’s Note

All claims expressed in this article are solely those of the authors and do not necessarily represent those of their affiliated organizations, or those of the publisher, the editors and the reviewers. Any product that may be evaluated in this article, or claim that may be made by its manufacturer, is not guaranteed or endorsed by the publisher.

## Footnotes

1
https://github.com/marcelm/cutadapt
